# A survey of *Babesia* spp. and *Hepatozoon* spp. in wild canids in Israel

**DOI:** 10.1186/s13071-018-2715-x

**Published:** 2018-03-20

**Authors:** Maayan Margalit Levi, Yaarit Nachum-Biala, Roni King, Gad Baneth

**Affiliations:** 1 Koret School of Veterinary Medicine, The Hebrew University of Jerusalem, Rehovot, Israel; 2 Israel Nature and Parks Authority, Jerusalem, Israel

**Keywords:** Golden jackal, *Canis aureus*, Red fox, *Vulpes vulpes*, *Hepatozoon canis*, “Babesia vulpes”, *Babesia lengau*

## Abstract

**Background:**

*Babesia* spp. and *Hepatozoon* spp. are apicomplexan parasites that infect a variety of animals, including canids. Their life-cycle includes an invertebrate hematophagous vector as a definitive host and vertebrates as intermediate hosts. The aims of this study were to investigate the prevalence and risk factors for *Babesia* spp. and *Hepatozoon* spp. infections in wild golden jackals (*Canis aureus*) and red foxes (*Vulpes vulpes*) in Israel and to compare spleen with blood sample polymerase chain reaction (PCR) for the detection of infection.

**Results:**

Blood and spleen samples from 109 golden jackals and 21 red foxes were tested by PCR for the detection of *Babesia* spp. and *Hepatozoon* spp. using primers for the *18S* ribosomal (r) RNA gene. *Hepatozoon canis* was detected in 50/109 (46%) of the jackals and 9/21 (43%) of the foxes. “Babesia vulpes” (the *Babesia microti*-like piroplasm) was detected in 4/21 (19%) of the foxes and in none of the jackals. A previously unknown genotype termed *Babesia* sp. MML related to *Babesia lengau* (96–97% identity) was detected in 1/109 (1%) of the jackals and 4/21 (19%) of the foxes. Further characterization of this genotype carried out by PCR of the rRNA internal transcribed spacer 2 (ITS2) indicated that it had only 87% identity with the *B. lengau* ITS2. Sex (male or female), age (juvenile or adult) and geographic zone (North, Central or South Israel) were not found to be significant risk factors for these protozoan infections. The prevalence of “B. vulpes” and *Babesia* sp. MML infections was significantly higher in foxes compared to jackals (*χ*^2^ = 15.65, *df* = 1, *P* < 0.005), while there was no statistically significant difference in the rate of *H. canis* infection between these two canid species. A fair agreement beyond chance between identification in the blood and spleen of *H. canis* was found in 21 animals from which both blood and spleen samples were available (k = 0.33).

**Conclusions:**

This study describes a high prevalence of *H. canis* infection in foxes and jackals and is the first report of “B. vulpes” infection in Israel, an area where *Ixodes* spp. are rare. It describes infection with a previously unknown genotype of *Babesia* related to *B. lengau* from Africa.

## Background

Blood parasites of the genera *Babesia* and *Hepatozoon* are apicomplexan protozoans which infect a large variety of animals, including canids [1–4]. Their life-cycles comprise an invertebrate hematophagous vector as a definitive host and vertebrates including domestic dogs and wild canids as intermediate hosts. *Babesia* spp. are transmitted by the saliva of ixodid ticks, infect erythrocytes and cause babesiosis, which is characterized clinically mainly by hemolysis and anemia. Infection with *Babesia* spp. can be sub-clinical, moderate or severe and cause potentially fatal disease [3, 5].

*Hepatozoon* spp. have a diverse range of vertebrate and invertebrate hosts with specific hematophagous invertebrate vectors that infect vertebrate hosts by ingestion of the invertebrate host containing mature *Hepatozoon* spp. oocysts. Some *Hepatozoon* spp., infect leukocytes of mammals, whereas other species adapted to lower vertebrates mainly infect the erythrocytes of their vertebrate hosts. *Hepatozoon* spp. can cause sub-clinical infections, or induce a mild disease, and some species such as *Hepatozoon americanum* may cause severe and fatal disease in their hosts [1, 2, 6].

Domestic dogs and wild canid species are often infected by the same species of *Babesia* and *Hepatozoon*, and from the evolutionary standpoint it has been suggested that these protozoan pathogens have been transferred to the domestic dogs from their wild canid species relatives. It is, therefore, likely that domestic dogs and wild canids living in the same geographical regions may share the same *Hepatozoon* and *Babesia* spp. infections [2, 7]. However, different habitats of domestic and wild canids, the specific susceptibility of the canid host species to the pathogen, and exposure to a different spectrum of hematophagous ectoparasite vectors are among the factors that may influence the probability of pathogen infection in domestic dogs and wild canid populations. Although there are several studies on the prevalence of *Hepatozoon* and *Babesia* species in wild canids from different parts of the world [8–13], in Israel there is little information on infection with these parasites in wild canids [14]. Hence, the aim of this study was to investigate the prevalence and risk factors for *Babesia* spp. and *Hepatozoon* spp. infections in the wild golden jackals (*Canis aureus*) and red foxes (*Vulpes vulpes*) in Israel.

As the spleen has a major role in removing bacterial and protozoal pathogens from the blood and often harbors protozoal infections in canids [15, 16], another aim of this study was to compare spleen with blood polymerase chain reaction (PCR) for the detection of *Babesia* and *Hepatozoon* infections. Knowing which tissue to choose for the best likelihood of parasite detection would be helpful for future studies.

## Methods

### Jackals and foxes

Spleen and blood samples were collected from golden jackals and red foxes by wardens of the Israel Nature and Parks Authority as a part of a national study on the prevalence of leishmaniosis in wildlife animals. Trapping of animals was performed by wardens of the Israel Nature and Park Authorities with an approved permit from this agency and the study was conducted adhering to the Hebrew University’s guidelines for animal husbandry and use of animals in research. The data collected on the animals included the location of trapping in North, Central or South Israel, sex, and age (Table 1).

**Table 1:** Numbers of golden jackals and red foxes included in the study, sample types examined, region or origin, gender, age and infection values

	Golden jackal	Red fox
Sample number and type		
Blood	65	17
Spleen	63	9
Blood and spleen	19	5
Total	109	21
Region in Israel (%)		
North	63 (58)	8 (38)
Center	20 (19)	9 (43)
South	21 (19)	2 (10)
Unknown	5 (4)	2 (9)
Gender (%)		
Male	52 (48)	9 (43)
Female	39 (36)	8 (38)
Unknown	18 (16)	4 (19)
Age (%)		
Juveniles	14 (13)	2 (10)
Adults	24 (22)	3 (14)
Unknown	71 (65)	16 (76)
Infection rates (%)		
*Hepatozoon canis*	50 (46)	9 (43)
"Babesia vulpes"	4 (19)	0 (0)
*Babesia* sp. MML	1 (1)	4 (19)

### DNA extraction, PCR and sequencing

DNA was extracted from blood and spleen samples with a commercial purification kit (Illustra Blood GenomicPrep Mini Spin Kit; GE Healthcare, Buckinghamshire, UK), according to the manufacturer’s instructions and a series of polymerase chain reaction (PCR) assays was run to identify *Babesia* spp. and *Hepatozoon* spp. infections. DNA from the blood of a laboratory-bred piroplasmid-free dog and from a dog naturally infected with *Babesia vogeli* and a dog naturally infected with *Hepatozoon canis* were used as negative and positive controls, respectively, and run with each reaction. PCR using the piroplasmid forward (5′-CCA GCA GCC GCG GTA ATT C-3′) and piroplasmid reverse (5′-CTT TCG CAG TAG TTY GTC TTT AAC AAA TCT-3′) primers was performed to amplify an approximately 360 base pair (bp) partial sequence of the *18S* ribosomal (r) RNA gene of piroplasm and *Babesia* spp. [17, 18].

The PCR was run using 1 μl primers (10 μM), 20 μl Ultra-Pure Water (UPW) and 3 μl DNA. The following conditions were used for amplification: 94 °C for 3 min; 35 cycles of 94 °C for 30 s, 64 °C for 45 s, and 72 °C for 30 s; and 72 °C for 7 min. The PCR was performed using the Syntezza PCR-Ready High Specificity kit (Syntezza Bioscience, Jerusalem, Israel).

A second PCR was performed on samples positive by the piroplasmid PCR for *Babesia* spp. to amplify a longer sequence of the *Babesia 18S* rRNA gene. The primers used to amplify a longer *18S* rRNA DNA sequence were 522F forward (5′-GTT GAT CCT GCC AGT AGT-3′) and 1661R reverse (5′-AAC CTT GTT ACG ACT TCT C-3′) which amplify 1700 bp of the *Babesia* spp. *18S* rRNA gene [19]. The PCR was run using 1 μl primers (10 μM), 20 μl UPW and 3 μl DNA. The following conditions were used for amplification with the 522F/1661R primers: 95 °C for 5 min; 35 cycles of 95 °C for 60 s, 59 °C for 60 s, and 72 °C for 60 s; and 72 °C for 5 min.

An additional PCR was performed to target the *Babesia* ribosomal operon internal transcribed spacer region 2 (ITS2) for some of the *Babesia*-positive samples which yielded a DNA sequence that did not match with a known species. This PCR was performed using primers FOR7 (5′-AGC CAA TTG CGA TAA GCA TT-3′) and REV7 (5′-TCA CTC GCC GTT ACT AGG AGA-3′) [20]. The following conditions were used for amplification: 95 °C for 5 min; 35 cycles of 95 °C for 60 s, 59 °C for 60 s, and 72 °C for 60 s; and 72 °C for 5 min.

PCR products were separated by electrophoresis in 1.5% agarose gel stained with ethidium bromide. Amplified samples were purified using EXOSAP (Exo-SAP, NEB; New England Biolabs, Inc. Ipswich, MA, USA), and sequenced from both sides at the Center for Genomic Technologies, Hebrew University of Jerusalem, Israel. The sequences were evaluated using the Chromas Lite software (Technelysium Pty Ltd., Brisbane, Australia) and compared to sequences deposited in GenBank using the Basic Local Alignment Search Tool (BLAST). A result was considered positive for a certain pathogen if it was the first match by BLAST and had at least a 97% identity with a known GenBank accession.

### Phylogenetic analysis

Phylogenetic analysis was performed using the Molecular Evolutionary Genetics Analysis software MEGA, version 6 [21]. The Maximum Likelihoood method was used to infer tree topology. Confidence values for individual branches of the resulting tree were determined by a bootstrapping analysis in which a 70% or higher value was considered significant.

### Statistical analysis

Data were analyzed using the Chi-square or Fisher's exact tests. Exact binomial 95% confidence intervals (CI) were established for proportions. A *P*-value < 0.05 was considered statistically significant. Comparison of spleen with blood sample PCR for the detection of *Hepatozoon* infections was calculated with the Cohen’s kappa coefficient (k). Analyses were done using the SPSS® 21.0 statistics software (IBM; Armonk, New York, USA).

## Results

Spleen and/or blood samples were collected from 109 golden jackals (*C. aureus*) and 21 red foxes (*V. vulpes*) (Table 1). The jackals originated from North (*n* = 63, 58%), Central (*n* = 20, 19%), and South Israel (*n* = 21, 19%). No information was available on the location of five jackals. Fifty-two (48%) of the jackals were males, 39 (36%) were females, and gender was not recorded for 18. Some of the jackals were classified as juveniles (*n* = 14, 13%) or adult (*n* = 24, 22%) while there was no information on the age of 71 of the jackals.

**Table 2:** *Babesia* spp. DNA sequences from red foxes and a golden jackal from the current study used for the *Babesia 18S* rRNA gene phylogenetic analyses and their closest GenBank matches

Sample number	Species	Host	GenBank ID	Size of *18S* rRNA fragment (bp)	Closest GenBank entry/origin	Percent identity
910	“Babesia vulpes”	*Vulpes vulpes*	KJ871348	302	MF040155/ “Babesia vulpes”/ Turkey	99
917	“Babesia vulpes”	*Vulpes vulpes*	KJ871349	307	MF040155/ “Babesia vulpes”/ Turkey	99
1061	“Babesia vulpes”	*Vulpes vulpes*	KJ871350	303	MF040155/ “Babesia vulpes”/ Turkey	99
910	“Babesia vulpes”	*Vulpes vulpes*	KJ871351	1638	AY534602*/ Babesia* sp. “Spanish dog”/ Spain	99
912	*Babesia* sp. MML	*Canis aureus*	KJ956779	314	KM025199/ *Babesia* sp./ South Africa	98
1017	*Babesia* sp. MML	*Vulpes vulpes*	KJ956780	313	KF270672/ *Babesia lengau*-like/ Zambia	97
913	*Babesia* sp. MML	*Vulpes vulpes*	KJ956781	312	KF270672/ *Babesia lengau*-like/ Zambia	97
913	*Babesia* sp. MML	*Vulpes vulpes*	KJ956782	1604	GQ411417/ *Babesia lengau*/ South Africa	97
1017	*Babesia* sp. MML	*Vulpes vulpes*	KJ956783	1639	GQ411417/ *Babesia lengau*/ South Africa	97

The foxes originated from North (*n* = 8, 38%), Central (*n* = 9, 43%), and South Israel (*n* = 2, 10%), and no information was available for two animals. Nine (43%) of the foxes were males and 8 (38%) were females while gender was not recorded for 4 (19%) the foxes. The foxes were classified as juveniles (*n* = 2, 10%) or adult (*n* = 3, 14%). No information was available on the age of 16 of the foxes.

### Prevalence of infection

*Hepatozoon canis* infection was identified in 50 out of 109 of the golden jackals (46%; CI: 35–55%) and in nine of 21 red foxes (43%; CI: 20–60%). “Babesia vulpes” (syns *Babesia microti*-like piroplasm, *Babesia* cf. *microti*, “Theileria annae”) was identified in 4 of 21 red foxes (19%; CI: 1–37%) and in none of the jackals. *Babesia* sp. MML (after the initials of the student who is the first author of this manuscript), a previously unknown *Babesia* genotype related to *Babesia lengau* (96–97% identity by *18S* rRNA gene sequence comparison) and *B. lengau*-like sequences (97% identity) (Table 2) was identified in 1 of 109 golden jackals (1%; CI: 0–3%) and in 4 of 21 red foxes (19%; CI: 1–37%). However, further genetic characterization by PCR of the ITS2 indicated that it had only 87% identity with the *B. lengau* ITS2 (Table 3).

**Table 3:** DNA sequence analysis of the *Babesia* sp. MML genotype ITS2 fragments, their GenBank accession numbers and closest GenBank entry match

Sample number	Species	Host	GenBank ID	Size of ITS-2 fragment (bp)	Closest GenBank entry/origin	Percent identity
1017	*Babesia* sp. MML	*Vulpes vulpes*	KR709304	323	KF510019/ *Babesia lengau*-like/ Zambia	87
912	*Babesia* sp. MML	*Canis aureus*	MG461685	444	KF510019/ *Babesia lengau*-like/ Zambia	87
1087	*Babesia* sp. MML	*Vulpes vulpes*	MG461686	445	KF510019/ *Babesia lengau*-like/ Zambia	87

### Comparison of blood and spleen infection

Altogether there were 65 blood and 63 spleen samples from jackals with 19 out of a total of 109 jackals from which both blood and spleen samples were taken. In addition, there were 17 blood and 9 spleen samples from foxes with 5 out of a total of 21 foxes which provided both blood and spleen samples. *Hepatozoon canis* was detected in 32/65 (49%) of the jackal blood samples and in 23/63 (37%) jackal spleen samples with a prevalence of 5/19 (26%) in jackals from which both blood and spleen were available. It was detected in 5 of 19 (26%) foxes with blood samples available and in 6 of 9 (67%) foxes from which the spleen was available. “Babesia vulpes” was identified only in foxes and found in 4/17 (26%) of the fox blood samples and in none of the 5 fox spleen samples. *Babesia* sp. MML was detected in 1/65 (2%) jackal blood samples and in no jackal spleens, while it was found in 3/17 (18%) fox blood samples and 2/5 fox spleens (40%). One of 5 foxes (20%) which had both blood and spleen samples was positive in both organs. The level of agreement between detection in the blood and spleen was calculated for *H. canis* only due to the small sample size of the other pathogens and was based on using 21 animals who had both blood and spleen available and of which 15 were positive in at least one tissue. Cohen’s kappa coefficient was 0.33 (CI: 0.06–0.60) with a fair level of agreement, between spleen and blood sample PCR for the detection of *H. canis* [22].

### Influence of host species, age, gender and geographical zone on infection

The effect of host species (*C. aureus* vs *V. vulpes*) was statistically significant (*χ*^2^ = 15.65, *df* = 1, *P* < 0.005) for “B. vulpes” and for *Babesia* sp. MML which were more prevalent in red foxes, but not for *H. canis* (*χ*^2^ = 0.065, *df* = 1, *P* = 0.799) found in similar prevalences in both jackals and red foxes. The effects of gender on the positivity for “B. vulpes” and *Babesia* sp. MML was statistically insignificant (*χ*^2^ = 1.675, *df* = 1, *P* > 0.05). The effect of age and gender on the positivity of *H. canis* were also statistically insignificant (*χ*^2^ = 3.79, *χ*^2^ = 0, respectively, *df* = 1, *P* > 0.05). There was not enough information to calculate the effect of age on positivity for “B. vulpes” and *Babesia* sp. MML. The effect of the geographical zone (North, Central and South Israel) on the positivity for “B. vulpes”, *Babesia* sp. MML and for *H. canis*, was statistically insignificant (*χ*^2^ = 2.04 for *Babesia* spp. and *χ*^2^ = 4.224 for *H. canis*; *df* = 2, *P* > 0.05). However, for “B. vulpes” and for *Babesia* sp. MML, no positive animals were identified in the South of Israel, whereas in Central Israel one and two of 29 animals were positive for these *Babesia* spp., respectively, and in North Israel four and two of 71 animals were positive, respectively.

### Genetic and phylogenetic analysis

**Fig. 1:**
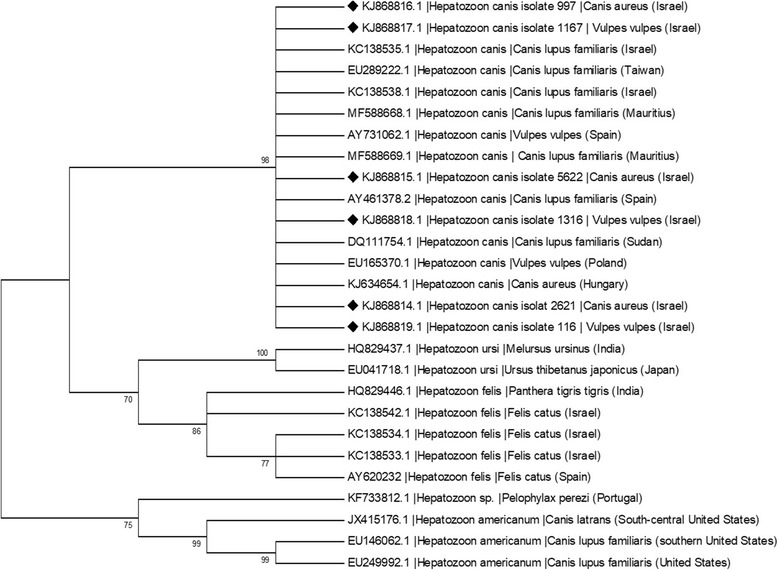
Phylogenetic relationship of *Hepatozoon canis* detected in this study to other *Hepatozoon* spp. based on a 325 bp partial sequence of the *18S* rRNA gene. The evolutionary history was inferred by using the Maximum Likelihood method based on the Hasegawa-Kishino-Yano model [47]. Sequences are presented by GenBank accession number, host species and country of origin. The diamond signs indicate the sequences derived from this study. The bootstrap consensus tree inferred from 1000 replicates [48] is taken to represent the evolutionary history of the taxa analyzed [48]. Branches corresponding to partitions reproduced in less than 70% bootstrap replicates are collapsed. The percentage of replicate trees in which the associated taxa clustered together in the bootstrap test (1000 replicates) are shown next to the branches [48]. Initial trees for the heuristic search were obtained automatically by applying Neighbor-Join and BioNJ algorithms to a matrix of pairwise distances estimated using the Maximum Composite Likelihood (MCL) approach, and then selecting the topology with superior log likelihood value

The positive PCR products from all reactions were sequenced to provide an accurate identity by comparison to GenBank accessions. All *H. canis* sequences were identical to *H. canis* sequences deposited in GenBank (MF588668, MF588669) with identity levels of 99–100%. Three *H. canis* sequences from golden jackals from this study were deposited in GenBank (KJ868814-KJ868816) and an additional three sequences from red foxes were also deposited (KJ868817-KJ868819) (Table 4). A phylogenetic analysis (Fig. 1) based on 325 bp partial sequences of the *18S* rRNA gene of *Hepatozoon* created using the Maximum Likelihood method based on the Hasegawa-Kishino-Yano model indicated that the six sequences deposited in GenBank clustered with a high bootstrap value with other *H. canis* sequences from domestic dogs, golden jackals and red foxes from Europe, Africa and Asia. The *H. canis* sequences clearly clustered separately from *H. americanum*, *Hepatozoon felis* and *Hepatozoon ursi* sequences.

**Table 4:** *Hepatozoon canis* DNA sequences from golden jackals and red foxes from the current study used for the *Hepatozoon* partial *18S* rRNA gene phylogenetic analysis and their closest GenBank matches

Sample number	Species	Host	GenBank ID	Size of *18S* rRNA fragment (bp)	Closest GenBank entry/origin	Percent identity
2621	*Hepatozoon canis*	*Canis aureus*	KJ868814	331	MF588668/ *Hepatozoon canis/* Mauritius	99
5622	*Hepatozoon canis*	*Canis aureus*	KJ868815	337	MF588668/ *Hepatozoon canis/* Mauritius	100
997	*Hepatozoon canis*	*Canis aureus*	KJ868816	326	MF588668/ *Hepatozoon canis/* Mauritius	100
1167	*Hepatozoon canis*	*Vulpes vulpes*	KJ868817	325	MF588669/ *Hepatozoon canis/* Mauritius	100
1316	*Hepatozoon canis*	*Vulpes vulpes*	KJ868818	328	MF588668/ *Hepatozoon canis/* Mauritius	100
116	*Hepatozoon canis*	*Vulpes vulpes*	KJ868819	330	MF588669/ *Hepatozoon canis/* Mauritius	99

**Fig. 2:**
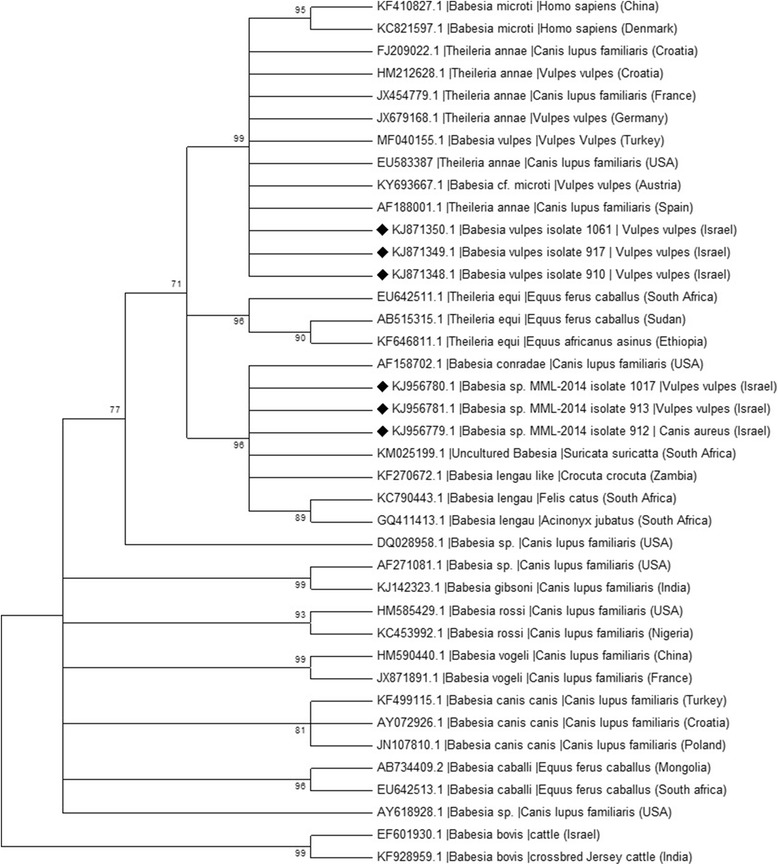
Phylogenetic relationship of *Babesia* spp. detected in this study to other *Babesia* spp. based on a 279 bp partial sequences of the *18S* rRNA gene. The evolutionary history was inferred by using the Maximum Likelihood method based on the Kimura 2-parameter model [47]. Sequences are presented by GenBank accession number, host species and country of origin. The diamond signs indicate the sequences derived from this study. The bootstrap consensus tree inferred from 1000 replicates [48] is taken to represent the evolutionary history of the taxa analyzed [48]. Branches corresponding to partitions reproduced in less than 70% bootstrap replicates are collapsed. The percentage of replicate trees in which the associated taxa clustered together in the bootstrap test (1000 replicates) are shown next to the branches [48]. Initial tree(s) for the heuristic search were obtained automatically by applying Neighbor-Join and BioNJ algorithms to a matrix of pairwise distances estimated using the Maximum Composite Likelihood (MCL) approach, and then selecting the topology with superior log likelihood value. A discrete Gamma distribution was used to model evolutionary rate differences among sites [5 categories (+G, parameter = 0.3763)]

**Fig. 3:**
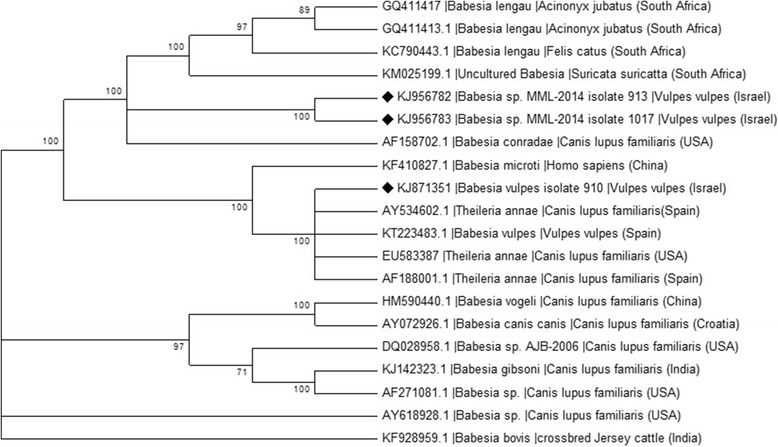
Phylogenetic relationship of *Babesia* spp. detected in this study to other *Babesia* spp. based on a 1490 bp long partial sequence of the *18S* rRNA gene. The evolutionary history was inferred by using the Maximum Likelihood method based on the Tamura 3-parameter model [47]. Sequences are presented by GenBank accession number, host species and country of origin. The diamond signs indicate the sequences derived from this study. The bootstrap consensus tree inferred from 1000 replicates [48] is taken to represent the evolutionary history of the taxa analysed [48]. Branches corresponding to partitions reproduced in less than 70% bootstrap replicates are collapsed. The percentage of replicate trees in which the associated taxa clustered together in the bootstrap test (1000 replicates) are shown next to the branches [48]. Initial tree(s) for the heuristic search were obtained automatically by applying Neighbor-Join and BioNJ algorithms to a matrix of pairwise distances estimated using the Maximum Composite Likelihood (MCL) approach, and then selecting the topology with superior log likelihood value. A discrete Gamma distribution was used to model evolutionary rate differences among sites [5 categories (+*G*, parameter = 0.2257)]. The rate variation model allowed for some sites to be evolutionarily invariable ([+*I*], 50.0746% sites)

“Babesia vulpes” sequences from this study were 99% identical to “T. annae”, *Babesia* sp. “Spanish dog” and “B. vulpes” sequences deposited in GenBank (KT580785, AY534602, MF040155, respectively). Four “B. vulpes” sequences from foxes in this study were deposited in GenBank (KJ871348-KJ871351) including a 1638 bp accession covering almost the whole *18S* rRNA gene (KJ871351). A phylogenentic tree constructed based on 279 bp partial sequences of the of the *18S* rRNA gene of *Babesia* using the the Maximum Likelihood method and the Kimura 2-parameter model (Fig. 2) showed that “B. vulpes” sequences from this study clustered together with *B. microti*-like piroplasm, “B. vulpes” and “*T. annae*” sequences from foxes and dogs from Europe, Turkey and North America, and separately from other piroplasm species that infect a variety of animal host species. Another phylogenetic tree based on longer *Babesia 18S* rRNA gene sequences of 1453 bp (Fig. 3) showed that when comparing longer sequences of the *18S* rRNA gene, “B. vulpes” from this study clustered with “T. annae” sequences from GenBank and away from *B. microti* and other *Babesia* spp. with even higher bootstrap values than in the analysis with short *18S* rRNA emphasizing the importance of performing the phylogenetic analysis with long DNA sequnences when available.

Sequences of the previously undescribed *Babesia* sp. MML from this study obtained when evaluating 312–314 bp segments of the *18S* rRNA amplified by the piroplasmid PCR were 97% identical to GenBank accession KF270672 of a *B. lengau*-like sequence from a hyena (*Crocuta crocuta*) in Zambia [13]. Two of the *Babesia* sp. MML sequences from red foxes were deposited in GenBank as accessions KJ956780 and KJ956781 and a sequence of this genotype from a golden jackal was deposited as KJ956779. The golden jackal sequence (KJ956779) was 98% identical to a *Babesia* sp. sequence (KM025199) from a meerkat (*Suricata suricatta*) from South Africa [23] (Table 2). These three *Babesia* sp. MML sequences from the current study clustered together in a phylogenetic analysis of short *18S* rRNA sequences (Fig. 2) with the *B. lengau*-like sequence from a hyena (*C. crocuta*) in Zambia and a *Babesia conradae* sequence from a domestic dog from California, USA. and close to a sub-clade with *B. lengau* sequences from a domestic cat and a cheetah (*Acinonyx jubatus*) from South Africa,

In the additional *Babesia* spp. phylogenetic tree based on longer *Babesia 18S* rRNA gene sequences of 1490 bp (Fig. 3), two long *18S* rRNA sequences of this new genotype from red foxes in Israel deposited in GenBank (KJ956782, KJ956783) clustered significantly separately from *B. lengau* sequences and from *B. conradae* and other *Babesia* spp.

**Fig. 4:**
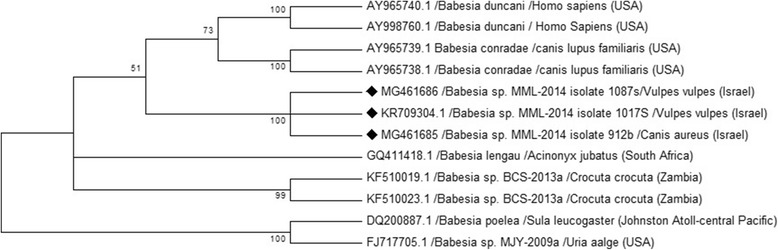
Phylogenetic relationship of *Babesia* spp. detected in this study to other *Babesia* spp. based on a 290 bp partial sequence of the ITS2 region**.** The evolutionary history was inferred by using the Maximum Likelihood method based on the Kimura 2-parameter model [47]. Sequences are presented by GenBank accession number, host species and country of origin. The diamond signs indicate the sequences derived from this study. The bootstrap consensus tree inferred from 1000 replicates [48] is taken to represent the evolutionary history of the taxa analysed [48]. Branches corresponding to partitions reproduced in less than 70% bootstrap replicates are collapsed. The percentage of replicate trees in which the associated taxa clustered together in the bootstrap test (1000 replicates) are shown next to the branches [48]. Initial tree(s) for the heuristic search were obtained automatically by applying Neighbor-Join and BioNJ algorithms to a matrix of pairwise distances estimated using the Maximum Composite Likelihood (MCL) approach, and then selecting the topology with superior log likelihood value. A discrete Gamma distribution was used to model evolutionary rate differences among sites [5 categories (+*G*, parameter = 0.5229)]

Further characterization of the new *Babesia* sp. MML carried out by amplification of the *Babesia* rRNA gene ITS2 region yielded two sequences from red foxes deposited in GenBank (KR709304, MG461686) and a sequence from a golden jackal (MG461685) which were only 87% identical to the *B. lengau*-like sequence from a hyena (*C. crocuta*) in Zambia (KF510019), which was the closest sequence to the *18S* rRNA gene sequences of the new genotype. These red fox and jackal sequences from Israel clustered in a phylogenetic tree based on 290 bp sequences of the ITS2 of *Babesia* spp. significantly seperate from *B. lengau*, *B. lengau*-like, *B. conaradae* and other species (Fig. 4). These results show that despite the 97% identity with the *B. lengau*-like *18S* rRNA sequence from Zambia and to *B. lengau*, the ITS2 region was considerably different from *B. lengau* and was likely to belong to a different *Babesia* sp.

## Discussion

Infection of wild canid species with tick-borne infections are common globally and vary regionally in the identity of the infectious agents and prevalence values. Although many studies have been published on *Hepatozoon* and *Babesia* spp. infection in red foxes, only a few publications have addresses these infections in golden jackals [24–27]. The high prevalence of *H. canis* in both jackals (46%) and foxes (43%) in Israel is in agreement with the high prevalence of this infection in domestic dogs [28] and the abundance of its vectors, *Rhipicephalus sanguineus* (*sensu lato*) and *Rhipicephalus turanicus* in this country [29]. Studies from European countries have also found high levels of *H. canis* infection in several countries, including in areas where *R. sanguineus* (*s.l.*) is not present, and therefore it was suggested that other vector ticks or different mechanisms of transmission such as transplacental transmission and carnivorism of infected hosts have a major role in the parasite’s transmission in foxes [30, 31]. It is probable that *H. canis* is well adapted to infecting foxes and jackals and is seldom associated with a severe disease as its high prevalence in these wild canids populations would not be expected if it was a common cause of severe disease.

“Babesia vulpes” has also been reported to be a frequent cause of infection in red fox populations in Europe and North America and recently also in the Asian part of Turkey [8–10, 32]. This is the first report of its presence in Israel, and there have not been any reports of its infection in domestic dogs, contrary to reports from countries such as Spain, Portugal and Croatia where infection of both dogs and red foxes have been reported [33–36]. The prevalence of “B. vulpes” infection (26%) found in red foxes in Israel in the current study based on a small animal sample is higher than found in Hungary (20%) [37] and lower than the 46%, 50% and 69% found in Austria, Germany and Portugal, respectively [9, 10, 12]. To date, infection of golden jackals with “B. vulpes” has only been reported previously once in 2/52 (4%) jackals from Romania [27]. “Babesia vulpes” has also been described in other canid species including the racoon dog (*Nyctereutes procyonoides*) in Austria [38], and the gray fox (*Urocyon cinereoargenteus*) in North America [8]. No “B. vulpes” was detected in jackals from Israel in this study and there have also not been any reports of domestic dog infection with this *Babesia* sp. in Israel to date. The tick vector or vectors of “B. vulpes” have not been incriminated yet, although several species of *Ixodes* have been implicated as possible vectors [39]. Interestingly, *Ixodes* spp. are rare in Israel and not likely to be the vectors of “B. vulpes” among red foxes in this country [40].

The detection of *Babesia* sp. MML in both foxes and jackals in this study is interesting since this *Babesia* genotype has not been reported before. *Babesia lengau* was initially described in cheetahs in South Africa and subsequently also associated with severe disease in two domestic cats from this country [41, 42]. Different *B. lengau*-like genotypes were detected in spotted hyenas (*C. crocuta*) and a lion (*Panthera leo*) in Zambia [13], brown hyenas (*Parahyaena brunnea*) and spotted hyenas in Namibia and South Africa [43] and lions in Botswana [44]. In addition, a hemolytic disease in sheep was attributed to a *B. lengau*-like piroplasm in northern Greece [45]. There appear to be a multitude of genotypes related to *B. lengau* described mainly from carnivores in southern Africa. We have characterized the new *Babesia* genotype from our study further by sequencing the ITS2 region and found it significantly different to *B. lengau* from cheetahs [41], and have therefore decided to term this genotype from foxes and jackals *Babesia* sp. MML.

*Hepatozoon canis* and *Babesia* sp. MML were found both in the blood and spleen of infected animals in this study, whereas “B. vulpes” was found only in the blood of foxes and not in the spleen. Other studies have shown that “B. vulpes” can be detected in the spleen [11]; however, two studies on foxes from Austria found that blood was more suitable for its detection that the spleen [10, 11], and blood is also preferred for “B. vulpes” detection in the fox over bone marrow [9]. While the current study found a higher prevalence of *H. canis* infection in the blood of jackals compared to their spleen, the spleen of foxes was a better tissue for the detection than blood in this study, in agreement with a study from western Austria [11].

The lack of significant difference in the presence of infection with the three parasites in jackals or foxes of different ages and gender is in agreement with findings from a study on vector-borne pathogens of foxes in Austria where *H. canis* and “B. vulpes” were studied [11]. Similar results on lack of influence of age and gender were also found for “B. vulpes” infection in foxes from Portugal [9]. When comparing to domestic dog infection with *H. canis*, in a survey of 694 dogs from Turkey, no difference in gender was found for dogs infected with *H. canis*; however, adult dogs were more frequently infected than young dogs [46]. In a study on Spanish dogs with clinical disease due to “B. vulpes”, no differences were noted in dog gender; however, dogs younger than 3 years of age were more susceptible to disease in comparison to older dogs [36]. These findings suggest that dogs and red foxes of both genders are probably equally susceptible to *H. canis* or to “B. vulpes” infection. The differences between foxes and dogs are related to age susceptibility with adult dogs more frequently infected with *H. canis* than young dogs whereas foxes of all ages were equally infected, and with young dogs showing more clinical disease with “B. vulpes” than old dogs. In contrast, the lack of age differences in fox infection may stem from differences in transmission pathways or degree of environmental exposure to the parasites and their vectors. The lack of significant differences in the prevalence of infection of the three parasites detected among jackals and foxes in North, Central and South Israel may be due to the small sample size, or to the fact that Israel is small (424 km from South to North and 114 km at its widest point). Distances between areas are short and without substantial boundaries which are difficult to cross, and therefore movement of wild canids from one area to another is likely.

This study had several limitations. It included a small number of red foxes and therefore might have missed some findings that could have been discerned from evaluating larger numbers of foxes and golden jackals. Furthermore, some information on age, gender and geographic location was missing for some of the animals. In addition, no co-infection was detected as the initial screening assay used and its sequencing directed to either *Hepatozoon* or *Babesia* infection, and led to additional PCRs as needed for characterization of the parasites identified initially.

This report represents the most southern point in which “B. vulpes” has been reported to date. The presence of “B. vulpes” in the fox population, which is known to be severely virulent to domestic dogs [33, 36], is a potential threat to the health of the canine population of Israel and the Middle East. Widespread *H. canis* infection of wild canids in Israel may present an important reservoir for infection of domestic dogs, as ticks feeding on jackals and foxes may infect dogs living in the same area because these wild canids often reach human dwellings in search of food and water. Furthermore, the newly detected *Babesia* sp. MML may prove to be pathogenic to wild canids as well as to domestic animals, and therefore requires further research and characterization.

## Conclusions

In conclusion, *H. canis* was found to infect close to half of the jackals and foxes included in the study, representing a high infection value with this pathogen in Israel. “Babesia vulpes” is reported for the first time in Israel and its infection may spread further to other canid species, and a new *Babesia* genotype was detected in both foxes and jackals and should be studied further to understand its possible pathogenicity and virulence to its hosts and other animals.

## Abbreviations

CI: 95% confidence interval
PCR: Polymerase chain reaction
rRNA: Ribosomal RNA
